# Spatializing 6,000 years of global urbanization from 3700 BC to AD 2000

**DOI:** 10.1038/sdata.2016.34

**Published:** 2016-06-07

**Authors:** Meredith Reba, Femke Reitsma, Karen C. Seto

**Affiliations:** 1 Yale School of Forestry & Environmental Studies, Yale University, New Haven, Connecticut 06511, USA; 2 Department of Geography, University of Canterbury, Christchurch 8020, New Zealand; 3 Yale School of Forestry & Environmental Studies, Yale University, New Haven, Connecticut 06511, USA

**Keywords:** Geography, Environmental social sciences, Environmental sciences, Anthropology

## Abstract

How were cities distributed globally in the past? How many people lived in these cities? How did cities influence their local and regional environments? In order to understand the current era of urbanization, we must understand long-term historical urbanization trends and patterns. However, to date there is no comprehensive record of spatially explicit, historic, city-level population data at the global scale. Here, we developed the first spatially explicit dataset of urban settlements from 3700 BC to AD 2000, by digitizing, transcribing, and geocoding historical, archaeological, and census-based urban population data previously published in tabular form by Chandler and Modelski. The dataset creation process also required data cleaning and harmonization procedures to make the data internally consistent. Additionally, we created a reliability ranking for each geocoded location to assess the geographic uncertainty of each data point. The dataset provides the first spatially explicit archive of the location and size of urban populations over the last 6,000 years and can contribute to an improved understanding of contemporary and historical urbanization trends.

## Background & Summary

Whether it is for timely response to catastrophes, the delivery of disaster relief, assessing human impacts on the environment, or estimating populations vulnerable to hazards, it is essential to know where people and cities are geographically distributed. Additionally, the ability to geolocate the size and location of human populations *over time* helps us understand the evolving characteristics of the human species, especially human interactions with the environment. Now that humans are predominantly an urban species, new questions arise as to how urban populations have changed over time. Where were cities of the past? What were the sizes of these urban populations and how were large and small cities geographically distributed? Many scholars question the sustainability of contemporary urbanization, arguing that the rates and scales of urbanization today present a new relationship between urbanization and the biosphere. However, in order to contextualize the current period of the urban demographic process and understand its potential future trajectories, we need data on long-term historical urbanization trends and patterns.

Currently, the only spatially explicit data on urban population with global coverage is the *United Nations World Urbanization Prospects*^1^. It is considered the most authoritative source of global urban population data and provides information on urban populations for major urban agglomerations around the world. The available data include latitude and longitude values for places with populations of 300,000 or more. However, these data are only available starting in 1950. Here, we provide the first spatially explicit, digital database of 6,000 years of urban settlements worldwide.

We developed the dataset using two principal sources: *Four Thousand Years of Urban Growth: A Historical Census*^2^, by historian Tertius Chandler and *World Cities: -3,000 to 2,000*^3^, by political scientist George Modelski. It is important to note that the dataset presented here is a spatial rendering of both Chandler’s and Modelski’s original datasets. We have not edited the population estimates themselves. We have harmonized the datasets and spatialized the data by providing latitude and longitude values for each settlement location. We have not extrapolated those city point locations to urban extents or city area estimates.

Our primary and original motivation for developing this dataset was to empirically test the oft-cited hypothesis that cities have historically developed in fertile agricultural areas. While the earliest record of cities traces their location to the Sumer region of Mesopotamia, or modern day Kuwait and Iraq, the hypothesis that cities in or near agricultural areas grow faster than cities located elsewhere has not been systematically tested. A long record of urban populations and their locations that spans at least a few hundred years would help establish the relationship between the growth of cities and geographic factors that shape or limit their development. Thus, this dataset was originally developed to test the relationship between historic patterns and rates of urban population growth and a city’s proximity to productive agricultural lands. However, this dataset has far broader applications than to test the relationship between the growth of cities and proximity to agricultural areas. A geolocated dataset of urban populations that spans several thousand years is a necessary first step to developing the ‘science of human settlements^4^.’ Although our dataset has a number of limitations including temporal and spatial sparseness and is far from comprehensive, researchers can use it to begin to explore the geographic evolution of urban settlements, the relationship between urban growth and resources, the geographic patterns of urban population growth, and long-term cycles of urban settlement growth and decline. In short, this dataset is a first step towards understanding the geographic distribution of urban populations throughout history and around the world.

Lewis Mumford, renowned urban historian, wrote in the foreword of *Four Thousand Years of Urban Growth,* ‘One of the most serious defects of historical urban studies is the lack of specific statistical information about the area, the density, and the population of cities^2^.’ In an attempt to fill this knowledge gap, Chandler devoted 30 years of his academic career to measuring and interpolating city-level population estimates globally through time. Chandler’s persistent and detail-oriented demeanor led to the development of one of the most complete, city-level population datasets today.

Chandler included only the largest cities for each time period: cities with populations over 20,000 from AD 800 to AD 1850 (excluding Asian cities which had a 40,000 population threshold for this period), and cities with populations over 40,000 after AD 1850 for all locations^2^. His methodology involved (1) collecting existing population and demographic information for the major cities of the world throughout history and (2) developing methods to estimate city-level population values where these population figures did not exist.

**Figure 1:**
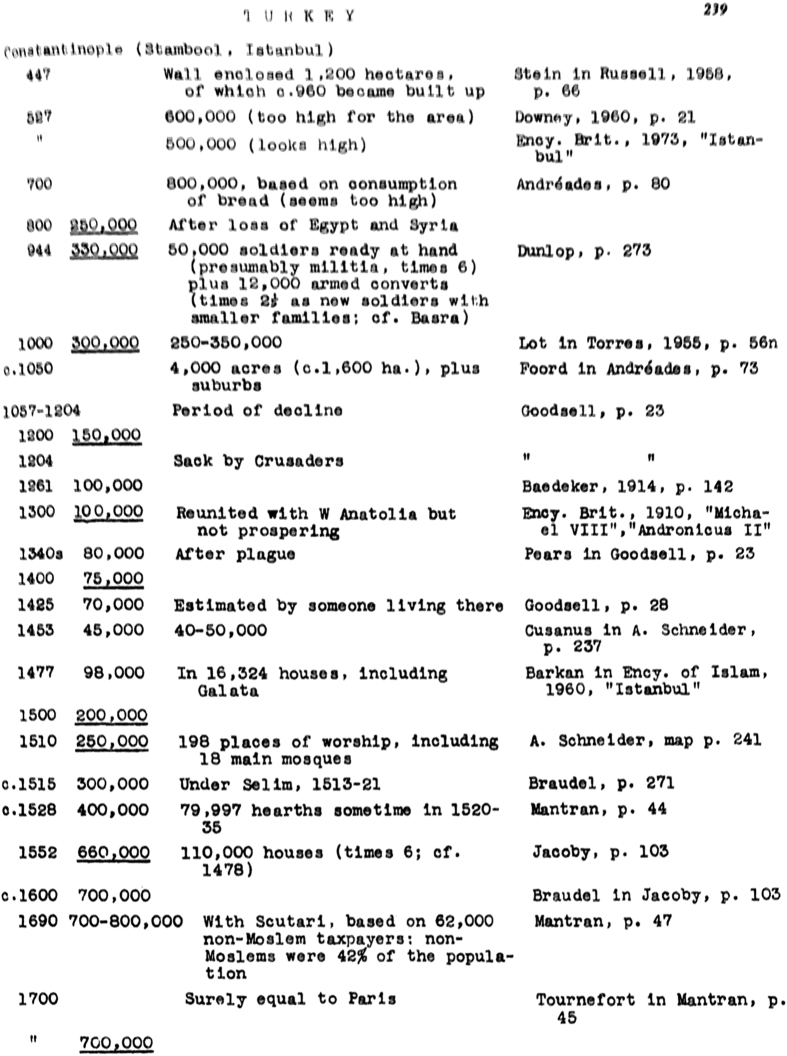
Chandler Text Sample. Sample page of Chandler’s *Data Sheets for Ancient Cities* section, which is the main section of both city population values and a description of their origin. The first column indicates the **year of the population estimate**, the second—**the estimate itself** (underlined figures are Chandler’s estimates), the third—the **derivation of the estimate**, and the fourth—the **citation information**. When interested in a particular city, time period, or population estimate, this detailed information permits a starting point for further in-depth analysis by referring to the citation sources.

Chandler utilized a wide range of sources to develop his volume: censuses, scholarly reports, travelers’ diaries, encyclopedias, gazetteers, travel guides, librarians, scholars, tax documents, street density, public bath counts, military records, disaster records of lives lost, and religious histories of cities^2^. From these sources, Chandler pieced together global city-level population estimates throughout time by first obtaining a demographic factor for a particular city, such as number of loaves of bread sold, and then applying a relevant multiplier. When possible, population estimates were cross-checked by multiple demographic factors. Occasionally, a rate of growth or decline based on a long record was also used to estimate a population value, such as for Kyoto, Japan^2^. Chandler also estimated population densities and population density factors for different walled cities. For example, the population density factor for a city with original or older walls was expected to be higher than the population density factor of the same city with expanded walls, since the wall expansion would be a result and indication of increasing population density^2,5^. Chandler’s estimate for each city for each time period relied on different methods. Many of these methods are discussed in Chandler’s text, as are his reference sources (e.g., see the fourth column of Fig. 1).

Although Chandler’s dataset is a milestone in the development of a historical urban population dataset, it has limitations, namely that it is temporally and spatially sparse. Temporally, there are gaps in measured or interpolated population values that are hundreds or thousands of years in duration. Spatially, only 1,599 geolocated cities are included in the dataset for the entire time period. Therefore, it is clear that this dataset alone is not a comprehensive global representation of urban populations through time. Rather, it highlights the urban populations and cities of importance during specific time periods.

The Modelski volume builds on and extends Chandler’s work. Modelski particularly focused on urban settlements from 3500 BC to 1000 BC and Sumerian, East Asian, and European-Mediterranean cities, which were underrepresented in Chandler’s original work^3^. Modelski used different minimum thresholds from Chandler for different eras to define a city:

- Ancient (3500 BC to 1000 BC):≥10,000 inhabitants
- Classical (1000 BC to AD 1000):≥100,000 inhabitants
- Modern (AD 1000 onward):≥1,000,000 inhabitants

**Figure 2:**
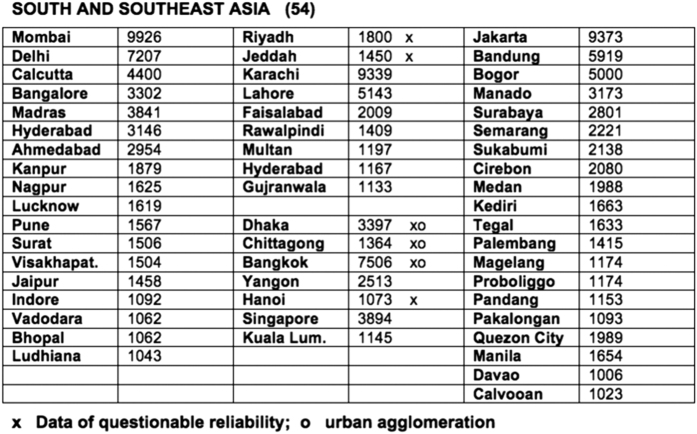
Sample Modelski Data Table. Sample table received directly from Modelski in Microsoft Word format. Cities are listed in bold type and are grouped by region (here, South and Southeast Asia). Population values are indicated in thousands. These tables were then transcribed into the Excel format highlighted in Supplementary Fig. 1 for further analysis.

Modelski also augmented Chandler’s dataset by including additional sources from archaeological records, historical works, and census data, while also selectively applying rank-order principles—also known as Zipf’s law or the power law. Zipf’s law states that when cities are ranked by size within a system, each city population is inversely proportional to its rank—meaning the second largest city is half as large as the top ranking city^3,6^. Primarily by referring to archaeological site assessments and calculating population-density factors, Modelski was able to extend Chandler’s original work by 1,475 years. He presented this information in descriptive text which identifies, explains, and quantifies changes in populations for urban settlements. Further, he recounted historic events leading to these changes and also included population estimates in tabular form (Fig. 2).

Both Chandler and Modelski used diverse sets of variables to determine their final city population counts. This variation reflects differences in urban definitions that persist today. Although the United Nations provide urban population estimates globally, the definition of urban and urban population varies by country. Some countries define an urban area using administrative or political boundaries. Other countries define an urban area as a place that exhibits certain characteristics, such as energy infrastructure or by characteristics of their populations, such as population size or population density. Norway defines urban as localities with 200 inhabitants. In contrast, Japan defines urban as places with at least 50,000 inhabitants. Even within a single country, definitions of urban can vary over time. For example, the definition of city and urban has changed many times in China during the 20th century, with earlier definitions based on designation of statutory cities and towns. More recent definitions of urban in China have focused on population densities and the presence of urban infrastructure.

Therefore, that Chandler and Modelski use different definitions and criteria for their estimates of urban is not surprising. While it can be argued that these differences in definitions preclude their direct comparison, much less integration, we posit that these multi-dimensional, multi-variable definitions of urban or city *improve* their characterization. It is well established that urban is a multi-dimensional concept, and that the city is defined in myriad ways by different disciplines and research communities. If there is some commonality to the conceptualization and definition of a city throughout history, it is that urban areas are often associated with a higher quality of life than non-urban places, manifested through more opportunities and more services.

In the urban literature, it is established that differences in urban definitions are not necessarily problematic. It is well understood that examining urbanization under a single development and population mobility model is inexact because it neglects the significant variations in context^7,8^. Similarly, when estimating population counts, distinct cities or time periods may be more suitable to particular demographic factors and methods. However, some suitable measures for comparison must exist. Chandler and Modelski attempt to cross-check these demographic factors and methods when possible. Undoubtedly, errors remain and a more rigorous system would strengthen these estimates.

Despite the varied source data and analytical methods used to create their two volumes, Chandler’s and Modelski’s works have been used for research extensively. Indeed, these two volumes are the only global-scale compendium of urban population pre-1950. According to the cited author search in Scopus accessed on March 15, 2016 *Four Thousand Years of Urban Growth* has been cited 199 times (including both the 1974 and 1987 editions of the text), and *World Cities* and a derivative peer-reviewed journal publication by Modelski^9^, have been cited more than 47 times. Both volumes have been used in myriad fields, including geography, economics, sociology, demography, history, anthropology, and the health sciences.

Chandler’s work has been frequently cited in reference to single urban population data points, such as Beijing being the only city in the world with a population of one million in AD 1800 (refs 10,11). Particular geographic locations for specific time periods have also been extracted from

Chandler’s data in order to study Liverpool as a resurgent city between AD 1700 and AD 1850 (ref. 12), to compare living conditions in small and large world cities from AD 1865–AD 1900 (ref. 13), or to measure how differences in economic institutions relate to levels of economic development^14^. The data have also been used to serve broader purposes. For example, a subset of Chandler’s time series has been used to study urban scaling laws^15^. Chandler’s data was also used to map civilizations and oikumenes—trade areas which are less tightly bonded societies than civilizations—in Aegean, Indic, Mexican, Peruvian, West African, Indonesian, Far Eastern, and Japanese civilizations to determine their decline phases^16^. Work discussing the potential quantity of communication within and through a city also sourced Chandler’s population counts to establish demographic size to determine the social structure of historic settlements^17^.

**Figure 3:**
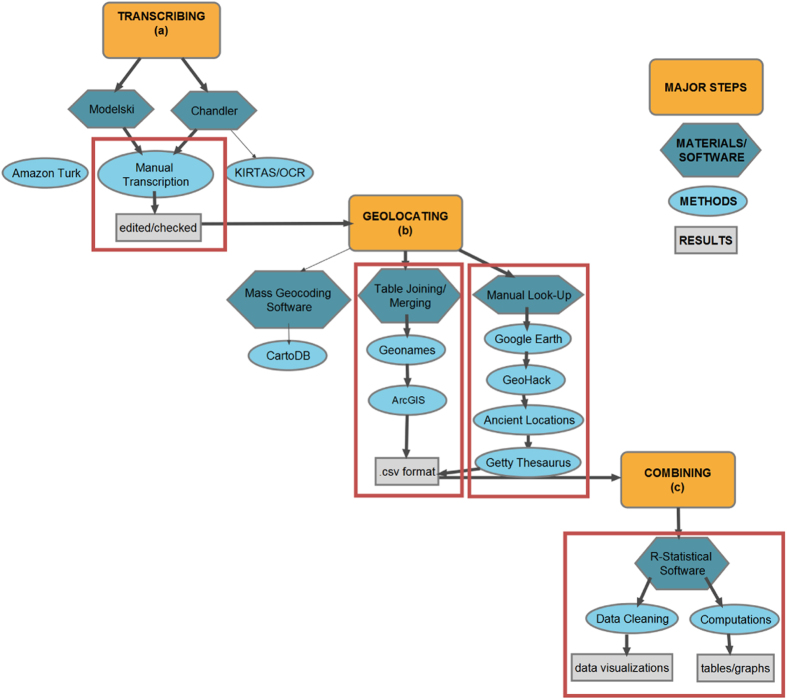
Study Schematic. Schematic overview of study design, highlighting the challenges of spatializing the dataset, as well as alternate methodologies. **Red** boxes indicate the resultant work flow path. Symbol shapes indicate: major steps (rounded rectangle), materials/software (oblong polygon), methods (oval), and results (rectangle). **Bold** arrows highlight flow path followed, non-bold arrows indicate attempted paths, no arrow signifies an alternate approach considered, but not attempted.

Figure 3 provides a schematic overview of our dataset construction and is referred to throughout the Methods section to clarify the dataset development process.

## Methods

### Transcription

**Figure 4:**
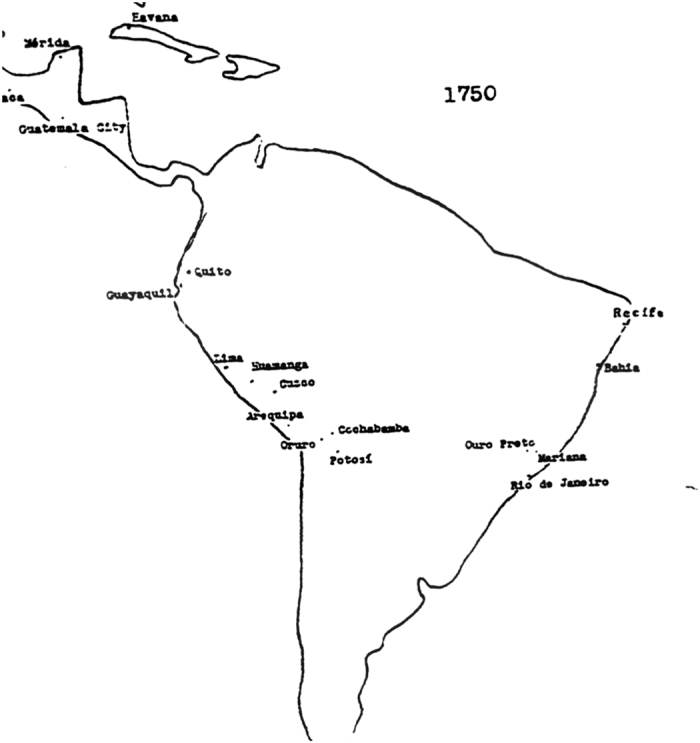
Sample Chandler Map. Continental map illustration from Chandler’s book, located in the *Continental Tables and Maps* section of the text. Although useful in locating some cities, the image quality of these maps is variable even in the original text and they provide approximate locations only.

Chandler’s book includes population data from 2250 BC to AD 1975 in various charts and tables. The book contains 656 9×5.5 inch pages and is divided into multiple sections, including *Sources and Methods*, *Continental Tables and Maps* (highlighting locations of major cities as illustrated in Fig. 4), *Data Sheets for Ancient Cities* (the main tables of the book shown in Fig. 1), *Tables of the World’s Largest Cities*, and *Whereabouts of Unfamiliar Cities*. Each page in the *Data Sheets for Ancient Cities* section (Fig. 1) contains a range of 15-30 data points per page. These pages are divided into four columns: (1) data year, (2) the population value (underlined values are Chandler’s estimates), (3) text describing the origin of the population estimate, and (4) citation information for each entry.

As with any digitization project, a significant component of the project is to convert the printed text—in this case a hardcover book—into digital format. There are several ways this task could be done. Because the Chandler book is 656 pages, its size warranted use of a *Kirtas* machine. A *Kirtas* machine uses optical character recognition (OCR) to convert printed text into an encoded format. An OCR system is able to convert text into a portable document format (pdf), which can be manipulated using a word processing program. This differs from a scanner, which converts print media to a picture that cannot be readily manipulated.

We had planned to use a *Kirtas* machine to convert the printed text to digital format. However, due to issues associated with the font of the printed book, which was not easily recognized by the *Kirtas* machine, and the variable quality of the printed pages, (e.g., Fig. 1), none of the OCR software we tested—*Microsoft One Note*, *Adobe Acrobat Pro*, and *Free OCR*—were able to accurately convert the printed text. After multiple attempts with the *Kirtas* machine, this approach was discarded and the text was manually transcribed into Microsoft Excel (Fig. 3). In total, 1,746 city locations were originally transcribed and checked twice by research assistants for transcription errors and accuracy. If entries did not match in all three cases, we referred back to the original documents for assessment and amendment. The final Chandler dataset contains 1,599 city locations, since some originally transcribed cities were later combined or were unable to be geocoded accurately.

We received Modelski’s dataset directly from the author in digital text format depicted in Fig. 2. The book itself, which consists of 245 pages, contains descriptive text recounting shifts in population values and their origins. We formatted these Microsoft Word tables into Excel tables using a similar format to the Chandler dataset. This format includes country names along the y-axis and time periods across the x-axis as depicted in Supplementary Fig. 1.

### Geolocation

Geocoding is the process of assigning geo-referenced coordinates, or longitude and latitude values, to a record to identify its location on Earth’s surface. It is often the first step in any spatial analysis when the data are not already geolocated^18^. Online geocoding platforms, such as *CartoDB* or *Google Places API* (Application Program Interface) can be used to process large amounts of data when the entries can be matched in batch-mode. This process allows all locations (up to a pre-determined limit for some geocoding services, such as 10,000 queries per day for *Google Places API*) to be submitted in a group batch, rather than interactively or individually^19,21^.

Geocoding or geolocating tools have been used most frequently and discussed in relation to medical field-based studies, such as public health or epidemiology^19–21^. In these studies, geocoding is often done at the individual address level. Geocoding at the address level allows for application of accuracy validation techniques and procedures since address locations can be checked by multiple geocoding services. When geocoding at the address level, accuracy can be measured by comparing the distances between geocoded points of different methods^22,23^.

Here, we geocoded population data for cities using a single, central latitude and longitudinal point with 2 to 8 significant figures depending on the geocoding database used. Urban extent data, or polygons defining the city boundary rather than city center points, are not included in our dataset due to a lack of available data. This lack of area extent information may limit the type of analysis possible using this dataset, but the point estimates of population size is a first step towards developing a more comprehensive dataset of urban extent. For example, users of this dataset could estimate area extents based on assumptions about population densities and land use, but this adds another level of uncertainty. Ultimately, the quality of the final geocoded dataset is partially determined by the quality and limitations of the original data.

**Figure 5:**
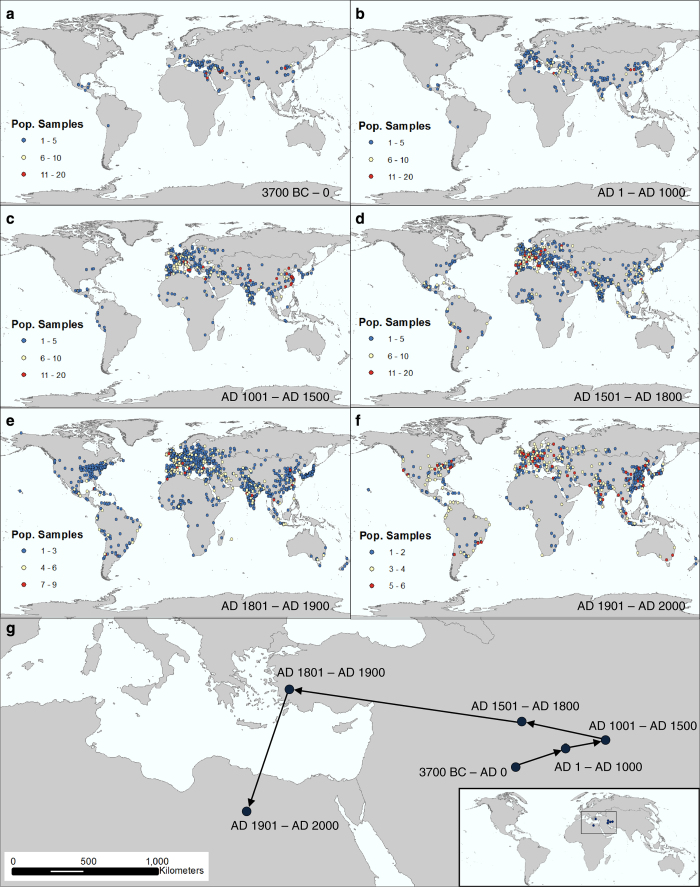
Spatial and Temporal Representation—Global View of Data Points. (**a**–**f**) illustrate both the spatial and temporal frequency of city-level population points for different time periods. (**a**–**d**) represent the pre-modern period, from 3700 BC–AD 1800, and use the same scale to measure frequency of data points per city. (**e**,**f**) represent the modern period and shorter time frame per period and therefore the frequency scale is shorter and separated into thirds. (**g**) illustrates global mean centers (GMCs) for the same time periods. Each GMC is weighted by city population for each data point and was calculated and is pictured in the Goode Homolosine projection.

After transcription of both datasets was complete, each city was geocoded, or assigned a corresponding longitude and latitude value. Spatial coverage of the entire dataset for six different time periods, accompanied by the frequency of data points per city for each period, is pictured in panels a-f of Fig. 5. Panel g shows population-weighted global mean centers (GMCs) for each of the six time periods. GMCs show the center of a region’s population at a given time point and can be useful to track human settlement patterns and shifts on a large, global scale over time. Here, we calculated the GMC by averaging all *x,y* coordinates (longitude, latitude) and *z*-cordinates (population) for each time period to determine the center of global population for each era. The results show an initial westward trend from the Mesopotamian origin. After the year AD 1500 this trend reverses and becomes westward. For the years between 2000 BC and AD 1000 where Modelski and Chandler sometimes recorded different population values for the same city/time period, we selected Modelski’s values, as his work focused on this ancient time period. The final dataset retains both Chandler’s and Modelski’s values for users to select at their discretion. However, it should be noted that the calculation of GMCs is one of many possible uses of this dataset.

Originally, we used geocoding software platforms, such as *CartoDB* (Fig. 3). However, due to the long timescale and global coverage of the data, there were changes in city names over time, as well as numerous similar city names across space. These similarities and name changes resulted in a many-to-many relationship between city names and city locations, and made it difficult to automatically and uniquely match the geographic location of a city.

Next, the *GeoNames* database was used to improve upon the *CartoDB* results. The database, or geographic gazetteer, derives its data from the *US Board of Geographic Names*, *Wikipedia*, the *Geospatial Intelligence Agency*, as well as using ‘ambassadors’ from over 70 countries representing over 250 regions, whose role is to discover potential global city/place location data sources such as military, governmental, educational, and mapping-based sources^24^. The continuously evolving result is a database of over 10 million geographical names which are all freely downloadable. *Geonames* contains a comprehensive list of cities with populations over 1,000 inhabitants, alternate city/locational names, type of location, and corresponding longitude and latitude. Data can be accessed through downloading a large text file or using their web services (API) for location matching. We downloaded a text document containing cities with over 1,000 inhabitants and then subsequently used this table in a table join, which involves merging two tables into a single table based on a common field (concatenated city/country name in this case), using *ArcGIS* software to match cities with their corresponding longitude and latitude values.

The *GeoNames* geographical gazetteer does have significant challenges and limitations. First, *GeoNames* does not provide coordinates for ancient locations or cities which have changed name over time. For this purpose both the *Ancient Locations*^25^ database of archaeological sites and the *Getty Thesaurus of Geographic Names*^26^ were used. *GeoNames* also contains only city point data, meaning it does not include urban extents. In a majority of cases, *GeoNames* determines these point data by calculating the centroid of a city area when available. However, some locations appear to use some other general point location within the urban extent^27^. This inconsistency most likely occurs as the result of user contributions to the database^27^. Toponyms, or multiple locations which share the same city name, are also challenging using table joining with *Geonames* and *ArcGIS* and can lead to multiple matches for the same city name.

Although more successful than the initial approach, cities remained either unmatched or matched to the incorrect geographic location. These errors arose due to varying original data quality in the Chandler and Modelski datasets. Considering the Chandler dataset, only approximately 50% of city names included a corresponding country name, although these other locations, which were included as tables highlighting the 75 largest cities of the world at specific time periods at the end of the text, could be cross-referenced with continental maps at the start of the book as shown in Fig. 4 or *Whereabouts of Unfamiliar Cities* section at the end of the text. However, as noted in Fig. 4, the image quality of these maps is quite variable, even in the original text. Modelski’s population tables also included only city names grouped by region/continent. Without a country name, it can be both challenging and time consuming to determine the correct geographic location due to toponyms. As a result, we added the most probable country names to all entries in the Chandler and Modelski datasets.

Alternate spellings, typographic errors, truncation of names to save characters, and city name changes over time can all serve to complicate and hinder the geocoding process. Inevitably, working with a larger, comprehensive dataset requires a significant amount of time and effort devoted to manual and programmatic data cleaning.

As a result, all entries were manually checked for accuracy. These errors were remedied by a one-by-one look-up in *Google Earth* and *Wikipedia’s GeoHack toolservers*^28^, which provides map sources and World Geodetic System 1984 (WGS 1984) coordinate system-based geolocations. *GeoHack* visually highlights specific locations through various global map services such as *GeoNames*, *Google Earth*, *Google Maps*, *OpenStreetMaps*, *MapQuest*, and *Bing Maps*. The *Ancient Locations* database, *Getty Thesaurus of Geographic Names*, and *GeoHack* database were also used for ancient city locations.

Less than 10% of the dataset was discarded as a result of unmatchable city locations due to changes in names and spellings. The final geocoding result had a 90% match rate. The standard minimum match rate necessary in determining a dataset to be spatially reliable is considered to be 85% for address-based data^29^.

Although a one-by-one look-up is a tedious process which can also lead to manual transcription errors, due to original data format challenges and omissions which prohibited accurate automated matching to large online databases, as well as the relatively small size of the dataset (1,741 city entries and 10,353 unique city/date/population values), this approach was undertaken. As a result, this dataset does not provide spatial confidence values—values which estimate the accuracy of the geocoded result. However, a geolocation reliability scale and rating was created and applied to the entire dataset based on the employed methods mentioned above. This rating scale is described and discussed in the Technical Validation section.

### Code availability

These three datasets were then combined using R statistical software to create one large population dataset spanning the years 3700 BC to 2000 AD. A sample script converts the three data sets (one from Chandler and two from Modelski—one each for ancient and modern time periods) from wide format into long format, standardizes the column names, and combines them into one large dataset^30^. A link to this script is included in the References section. However, many different approaches could be used to complete this task.

### Dataset development challenges and limitations

**Figure 6:**
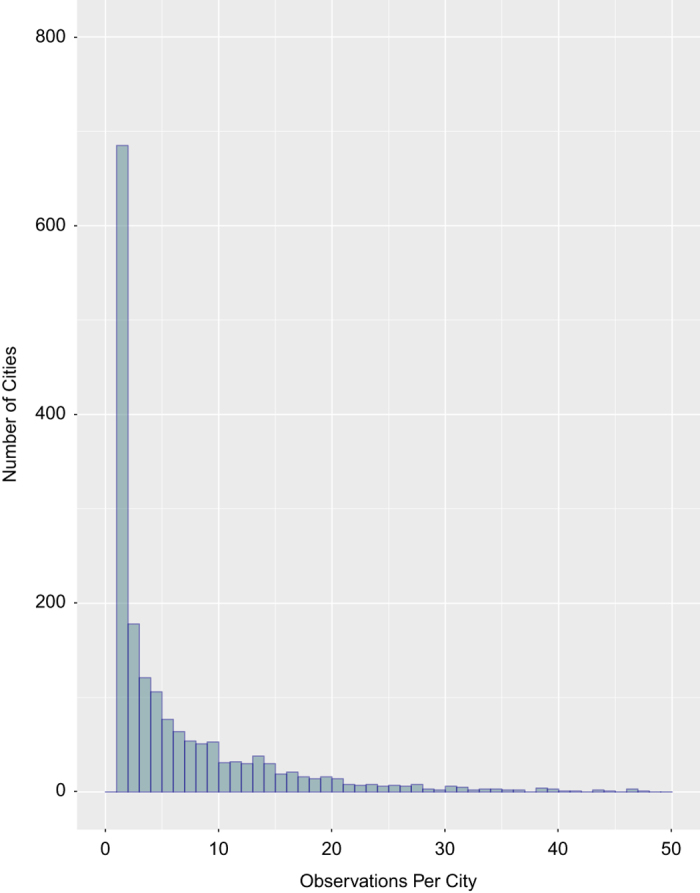
Temporal Representation—Frequency Histogram. Histogram reporting temporal frequency of data points for individual cities. Over 600 cities have only one population value for the entire dataset time period as indicated by the leftmost histogram bar, signifying the temporal sparseness of the dataset. Table 2 emphasizes the tail end of this distribution, highlighting cities with the highest frequency of population points through time.

Chandler’s data has a number of limitations. Despite Chandler’s accomplishment in creating the dataset, the resulting population data are temporally and spatially sparse as illustrated in Figs 5 and 6. Figure 5 provides a snapshot spatial view of all data points across the entire dataset for given time periods and highlights the number of population values for each city, while Fig. 6 illuminates the dataset’s temporal sparseness. As indicated by the leftmost bar in the histogram, over 600 cities of 1,741 original cities have only one city population value. As a result, temporal gaps in measured or interpolated population values can be hundreds or thousands of years in duration, especially before AD 1100. Data points are also sparse for South Asian, South American, North American, and African cities. These data alone are not accurate global representations of all population values through time. Rather, it highlights the population values of important global cities during important time periods. This fact limits the scope of analysis possible with this dataset.

Other limitations of Chandler’s work include both his definition of a city and his data interpolation methods. Chandler defines a city as ‘an urban area including suburbs lying outside of the municipal area, and omitting farmland lying within the municipality’ but follows this definition with the statement that suburb growth was not significant until 1850 (ref. 5). However, suburb, or peri-urban, growth has been documented prior to this point in history.

Cities and their boundaries and populations are constantly changing. Chandler’s early city population estimates used the spatial extent of the city to estimate the population using a common population density of the region and time period. However, these population densities shift with family and city structure^5^. Related archaeological and historical research helping to predict city size has also been significantly improved since Chandler published his dataset^5^.

It should also be noted that the definition of what constitutes an urban center during both different time periods and different regions is variable^31^. While Chandler and Modelski’s data provides general trends in population trajectories over time, these patterns can originate from quite different growth or abandonment narratives, as the true history of a settlement is complex^8,32^.

Since the publication of Chandler’s data in 1987, a number of scholars have proposed methods to improve the dataset. For example, Bairoch, Pasciuti, and Chase-Dunn have proposed and done work to progress city population coverage and accuracy of these historic city population estimates^5,33^. Pasciuti and Chase-Dunn proposed creating a new dataset of city populations for the Urbanization and Empire Formation Project in their report called *Estimating The Population Sizes of Cities*^5^. While Bairoch, who aimed to improve Chandler’s population estimates by also taking into consideration the land type within city walls (commercial, residential, gardens, or grazing), uninhabitable space within buildings, and the density of occupations, suggests increasing Chandler’s estimate of European city values by 15% and Latin American by up to 50% (refs 5,33).

It is also important to note that urbanization is not a linear process. There have been a number of historical events such as natural disasters (i.e., fires, earthquakes, droughts) and human conflicts (i.e., wars, invasions, colonialism, conflicts over natural resources) which have influenced human migration patterns and as a result, affected both settlement population counts and general urbanization trends. The objective of this work is not to reconcile these issues, but to spatialize the data so they are in a useable format for researchers, including historians, to critique these population estimates and analyze global urbanization trends.

Despite these challenges and shortcomings of the Chandler and Modelski data, the combined dataset presented here is a significant advance to the understanding of urbanization and cities through history. Although both the Chandler and Modelski volumes are available, they are not widely accessible nor readily usable in their tabular form. Both are available in print format only (although Modelski’s digital data was made available to the authors via personal communication), and the process of spatializing the data requires significant investment in human and technical resources, and also time. Thus, although this dataset does have limitations, it’s easy to access and useable format will allow it to be tested and examined by the wider research community, such as geographers, historians, archeologists, or ecologists. Spatializing and digitizing the dataset presents a new, improved basis for data dissection and visualization. Providing the data in this easily accessible and usable format also encourages a more rigorous critique by the scientific community of the population estimates. Left in their current forms, these valuable scholarly works remain in an impractical and less-usable format.

## Data Records

This dataset is stored in a spreadsheet format in.csv form. This format permits smooth, uncomplicated usage in statistical software packages such as R for further data compilation or analysis.

Three final documents were produced:

1. Chandler’s geolocated database (2250 BC–1975 AD)
2. chandler.csv (Data Citation 1)
3. Modelski’s Ancient geolocated database (3700 BC–1000 AD)
4. modelskiAncient.csv (Data Citation 2)
5. Modelski’s Modern geolocated database (2000 AD additions for major cities, all cities in China show 1990 census results)
6. modelskiModern.csv (Data Citation 3)

Both Chandler’s and Modelski’s datasets include the following columns/categories. A snapshot of this data is depicted in Supplementary Fig. 1:

- City
- OtherName
- Country
- Latitude
- Longitude
- Certainty measure
- Year with corresponding population value

The final, downloadable format of all 3 databases is also available from the Seto Lab: Urbanization and Global Change website at the following URL: www.urban.yale.edu/data.

## Technical Validation

### Reliability rating

Cities were also ranked based on the accuracy of their geocoded match. Reliability is a necessary factor to allow researchers and data users to be aware of the accuracy of the data and the subsequent analytical results^22^. Although cities in the *Data Sheets for Ancient Cities* section of Chandler’s book are linked with a country or continent of origin, Chandler also appended tables to the end of the text including the largest cities for a given time period. These cities do not report a corresponding country although some of these cities are indicated in the continental maps section of the book presented in Fig. 4 and *Whereabouts of Unfamiliar Cities* section at the end of the text. The final dataset digitized through the methods described here added the most probable country name determined through examining the maps at the beginning of the text, agreement between multiple geocoding techniques, and comparing the size of potential city matches with the Chandler’s population estimates for the given time period.

Because city names can often be repeated in different countries, also known as a typonym—such as Birmingham, England and Birmingham, USA—without a definitive country name, determining a city’s precise geolocation can be challenging and uncertain. City names also sometimes change throughout time, which adds another noteworthy challenge to geolocating historical locations. Therefore, alternate city names and spellings were also added to the database in the ‘OtherName’ category. Despite all these systems and precautions however, geocoding errors will still remain.

We established an accuracy ranking system for the entire dataset to test for consistency. This ranking system consists of a three-tiered structure with ranks of 1, 2, and 3. Cities holding a rank of 1 are considered most accurate, while those of rank 3 are least accurate. Ranks were determined through consistency of results. If a city’s geolocation was confirmed by three geocoding sources, we assigned an accuracy value of 1. Cities with matching results by two sources were assigned an accuracy value of 2, while cities that required numerous attempts to determine a potential geolocation and whose results were potentially more spurious were assigned a level 3 accuracy value. Less than 10% of the final dataset for which no geolocation could be determined was discarded. 75% of total cities in the dataset report an accuracy value of 1, 13% an accuracy value of 2, and 12% an accuracy value of 3.

This ranking system was created with the intention of allowing researchers to subset the dataset to the level of accuracy most suitable for each specific analysis. For example, for studies where the most exact locational information is required, cities with a certainty ranking of 2 or 3 could be rejected. Furthermore, improvement and enhancement of the dataset can be better targeted to those cities where geolocations are suspect—cities with an accuracy value of 2 or 3.

In addition to developing a reliability rating to provide technical validation of the dataset, multiple (3 total) transcribers were used to create the dataset. Disagreements in values were resolved by referring back to the original text and tables. An alternate to manually transcribing the dataset could be *Amazon Mechanical Turk*. *Amazon Mechanical Turk* is an agglomeration of ‘human intelligence tasks’ divided into sections for those who have work to be completed and those who are looking for workers. Using this alternative, one could have the dataset transcribed multiple times and then use statistical packages to test for errors or outliers.

### Temporal and spatial sparseness

Although the final dataset remains temporally and spatially sparse on a global level, certain regions contain a greater amount of data points for certain time periods. The tables below highlight years with the highest number of population points (Table 1) and cities with the top number of population data points through time (Table 2). Cities with the largest populations for specific time periods are also highlighted in Supplementary Tables 2 through 8, a comparison with UN urban population data for the year AD 2000 is included in Supplementary Table 8. The dataset can also be spatially subset for different countries or geographic regions such as Europe, India, or Asia, where data points are more spatially dense by spatially joining data points with continental maps. These subsets would allow for regional scale analyses with a higher frequency of population data points, both temporally and spatially.

## Usage Notes

Ultimately, we view this dataset as a beginning compilation of a richer historical, global, city-level population database. Despite the current spatial and temporal gaps, the dataset does provide a spatially explicit, long historical record of city population values at the global scale. At the current time no other geocoded dataset at this resolution exists. As a result, the dataset could be used as a foundation to build a richer record of urban population values through history, creating systematic, global population data to measure urban growth at a long timescale.

However, we caution potential dataset users of the following limitations and dataset details:

1. Some of Chandler’s data interpolation methods, including the development of his multipliers, use of soldiers to estimate city size, and his use of now outdated archeological records and methods, have been criticized as previously discussed^5^.
2. Alternate population estimates for the same locations and time periods exist and should be considered, especially those of Paul Bairoch^5,33^.
3. Spatially explicit city population estimates since 1950 also exist in the *United Nations World Urbanization Prospects*^1^. These data should also be considered. These data are easily accessible and use different parameters to limit an urban area (and are not compatible for a time series analysis without data modification due to different definitions of what constitutes a city in each of the different datasets). Therefore, they were not included here.
4. There is a discrepancy in the number of significant figures in the city’s latitude and longitude points due to the different geocoding services used to deliver latitude and longitude values. The number of significant figures ranges from 2 to 8.
5. Here, we only provide city point locations varying from 2 to 8 significant figures depending on the geocoding database used. We do not have data on urban extent for city locations. This lack of extent data may limit the type of analysis possible using this dataset.
6. City point locations differ between and within geocoding databases, such as *GeoNames*. For example, although *GeoNames* uses city point-level data, it appears as though sometimes this point is the geometrically calculated centroid, while in other cases, this point appears to be more generally located within the urban extent. This discrepancy is most likely due to the open user contributions inherent in the *GeoNames* database structure^24^.
7. Because multiple geocoding vendors/techniques needed to be used to match an acceptable city percentage and prevent/limit the number of non-matches, false positive matches (incorrect matches), and false negative non-matches (incorrect non-matches), we are unable to provide accompanying statistical analyses of experimental error and variation or spatial confidence values.
8. Although a reliability ranking scale has been created, some data points’ geographic location remains uncertain.
9. Chandler and Modelski’s definition of a city differ for the modern time period. Chandler considers suburbs outside the municipal area as part of the urban population from after 1962 (ref. 2); Modelski does not. Modelski’s modern era population (AD 2000) estimates use UN *2000 Demographic Yearbook* data for ‘cities proper—governed as one unit’ and do not show urban agglomeration population data unless no other data were available. All Chinese cities also show 1990 census results for Modelski’s AD 2000 population estimates. Please refer to Modelski’s text for individual city exceptions^3^. As a result, some city population values decline while others increase between Chandler’s last year of data (AD 1975) and Modelski’s modern data for the year AD 2000 as shown in Supplementary Tables 7 and 8. Year AD 2000 data is still useful as a yearly cross section. *UN Urbanization Prospects*^1^ data can also be considered for subsequent years after AD 1950.
10. Modelski occasionally designated a value of ‘E’ for a city population to represent a ‘population estimated to be 10,000 or more’ in his ancient dataset^3^. Sometimes these ‘E’ values occurred just before or after population counts an order of magnitude larger or more for the same city. Due to this large discrepancy, we chose to exclude ‘E’ value population estimates in the final dataset. As a result, 51 individual city-level population counts in Modelski’s Ancient Dataset were omitted (Data Citation 2).
11. Both the Chandler and Modelski datasets lack corresponding country names for many city locations. After entry number 790 in the Chandler dataset, original country names are very sporadic and Modelski’s population tables included only city names grouped by regions/continents^2,3^. As a result, we added the most probable country names to all city locations in the dataset. Modern country names according to the *United Nations World Urbanization Prospects* were used in all datasets.
12. Chandler and Modelski also include alternate names for some cities. These names are included in the final datasets in the ‘OtherName’ column. We also added additional alternate names, alternate spellings, and locational information such as state names for US cities to this category/column. Alternate city names are separated with a comma.
13. Our reliability ranking only ranks the certainty of geocoded city locations. To determine the reliability of Chandler’s and Modelski’s population estimates themselves please refer to the original texts^2,3^. Chandler’s personal estimates are underlined in the text, while Modelski marks ‘data of questionable reliability’ with an ‘x.’ We did not correct or validate population estimates in this dataset. We are aware population estimate errors may exist as a result. For example, the population estimate for Montevideo in AD 2000 of 13,303,000 is an order of magnitude too large, but we chose to leave the estimates unedited. We did not want to unsystematically begin editing population estimates, even those that are blatantly inaccurate. The purpose of our analysis was to spatialize the data, with the intention of allowing the users to validate the data to improve its accuracy and usability. Supplementary Table 8 compares Modelski’s estimates to *UN World Urbanization Prospects* data for the year AD 2000 to highlight discrepancies and variations.
14. Occasionally, there is a discrepancy in the reliability ranking for the same city during different time periods. This discrepancy is intentional. If a city used a questionable alternate name for a given time period, which was either given by the authors of the original datasets or determined by us, we adjusted the reliability ranking by one level (i.e., from a 1 to a 2 or 2 to a 3).
15. Periodically, a city name will change over time, such as Constantinople, Turkey to Istanbul, Turkey or Edo, Japan to Tokyo, Japan. In such known cases, the most recent city name is used in all datasets.
16. Spelling of primary city names in all 3 datasets has also been updated to match the UN database of city names whenever possible. We matched city names to both the *United Nations World Urbanization Prospects* tables for settlements with populations over 300,000 inhabitants and the *United Nations Code for Trade and Transport Locations* tables *(UN/LOCODE)* using the name without diacritics column. Some ancient cities and historical archaeological sites do not have modern day equivalents. In such cases, original names from the Chandler and Modelski datasets were retained.
17. We did not complete additional spatial analyses to further combine nearby city points that may be part of an urban agglomeration. Only alternate city names and city names that clearly shifted through time were combined. Users could carry out further proximity-based spatial analysis measures.
18. In the ancient time period, Chandler and Modelski’s population values sometimes differ for the same city during the same year. For example, Modelski cites Istanbul’s (Constantinople’s) population as 500,000 in AD 500, while Chandler cites the population as 400,000. Both values are retained in the final dataset, leaving the user to decide which value to select as most accurate. For all plots and tables displaying data for the years between 200 BC and AD 1000 where Modelski and Chandler recorded different population values for the same city and time period, we used Modelski’s values, as his work focused on this ancient time period.
19. Long time-scale, global analyses based on our dataset alone are difficult, due to the spatial and temporal sparseness of data points highlighted in Figs 5 and 6 and Tables 1 and 2.
20. Finally, we would like to remind users of the dataset that measuring population growth and population aggregates are only one component of the urbanization process.

Future work could augment the dataset by filling in population gaps using updated datasets, such as UN population datasets, archaeological data, or the work of Bairoch which has suggested systematically increasing the populations of European and Latin American cities in particular^1,5,33^. Inaccurate or missing geocoded locations could also be improved.

## Additional Information

**How to cite this article:** Reba, M. *et al.* Spatializing 6,000 years of global urbanization from 3700 BC to AD 2000. *Sci. Data* 3:160034 doi: 10.1038/sdata.2016.34 (2016).

## Supplementary Material



Supplementary Figures and Tables

## Figures and Tables

**Table 1:** Years recording the highest frequency of city population points.

**Year**	**No. of city points**
1900	1,094
1800	511
1850	484
1950	430
1975	393
1750	294
2000	293
1700	272
1600	238
1925	214
1500	203
1400	173
1300	172
1200	147
1851	116
1000	107
1801	91
1550	89
1650	89
1825	84
1450	83
1100	81
800	75
1250	74
1875	74
The full dataset could be subset by these denser time periods to determine trends over time.	

**Table 2:** Cities recording the highest frequency of population points through time.

**City**	**No. of years of population data**
Istanbul, Turkey (Constantinople)	47
Milan, Italy	46
Rome, Italy	46
Suzhou, China	46
Paris, France	44
Barcelona, Spain	43
Xian, China	43
Nanjing, China	41
Alexandria, Egypt	40
Beijing, China	39
Guangzhou, China	39
Naples, Italy	39
Aleppo, Syria	38
Cairo, Egypt	38
Chengdu, China	38
Hangzhou, China	38
Fuzhou, China	36
Messina, Italy	36
Lisbon, Portugal	35
Palermo, Italy	35
Delhi, India	34
Kaifeng, China	34
Venice, Italy	34
Damascus, Syria	33
London, England	33
The full dataset could be subset by these regions to increase the temporal frequency for time series based analyses.	
