# Supplementary material for: Spatializing 6,000 years of global urbanization from 3700 BC to AD 2000
Source: Sci Data. 2016 Jun 7;3:160034. doi: 10.1038/sdata.2016.34 (PMC4896125; doi:10.1038/sdata.2016.34)
Supplement: Supplementary Figures and Tables [file sdata201634-s2.pdf]

Table of Contents

Supplementary Figure 1……………………………………………………………1
Supplementary Table 2…..………………………………………………………….2
Supplementary Table 3…….……………………………………………………….2
Supplementary Table 4…..………………………………………………………….3
Supplementary Table 5…..………………………………………………………….3
Supplementary Table 6…….……………………………………………………….3
Supplementary Table 7…….……………………………………………………….4
Supplementary Table 8…..………………………………………………………….4

	A	B	C	D	E	F	G	H	I	J	K	L	M
1	City	OtherName	Country	Latitude	Longitude	Certainty	BC_3700	BC_3500	BC_3300	BC_3000	BC_2800	BC_2500	BC_2400
2	Eridu		Sumer	30.81584	45.9960703	1	6000	10000	10000	10000			
3	Uruk		Iraq	31.32222	45.636111	1		14000	40000	40000	80000	40000	30000
4	Larak	Kesh	Sumer	32.31274	45.660998	1		10000	10000	10000	40000	10000	10000
5	Nippur		Iraq	32.12694	45.2307853	1				10000	10000	20000	20000
6	Sippar		Sumer	33.05883	44.2521534	1							
7	Shuruppak		Sumer	31.77736	45.5097612	1					30000	30000	10000
8	Badtibira		Iraq	31.76667	46.004444	1							
9	Anshan		Iran	30.01202	52.4085766	1			10000	10000	10000	10000	10000
10	Nagar	Tell Brak	Syria	36.66739	41.0586539	1				20000	20000	15000	10000
11	Ur		Iraq	30.96266	46.10321	1					12000	10000	10000
12	Zabalam	Zabala	Iraq	31.74333	45.876667	2					10000	10000	10000
13	Kish		Sumer	32.55218	44.5856425	1					30000	20000	
14	Lagash		Iraq	31.4148	46.4098159	1						60000	30000
15	Girsu		Iraq	31.56003	46.1754283	1							40000
16	Nina	Surghul	Iraq	31.41139	46.407222	3							
17	Umma		Sumer	31.6211	45.9334459	1					20000	40000	40000
18	Adab		Sumer	31.94701	45.9684213	1					10000	20000	10000
19	Akshak		Sumer	33.9472	44.2447172	3						10000	20000

SUPPLEMENTARY FIGURE 1: Our Dataset Format: Example of excel formatting used to transcribe Chandler and Modelski data. City names run along the y-axis, while the x-axis includes: *other/alternate city names, country, latitude, longitude, certainty ranking* (explained in the *Technical Validation* section), and *years* - which contain population data. These .csv spreadsheets can be easily imported in R or other statistical software packages for further analysis.

Supplementary Tables 2-8: Largest cities (recording the highest population values) for the indicated time period.

Note: For the years between 2000 BC and AD 1000 (**Tables 2, 3,** and **4** below) where Modelski and Chandler recorded different population values for the same city/time period, we selected Modelski's values, as his work focused on this ancient time period. The final dataset retains both Chandler's and Modelski's values for users to select at their discretion.

Supplementary Table 2: Largest Cities in the Year 2000 BC

Largest Cities in the Year 2000 BC	Population
Memphis, Egypt	60,000
Girsu, Iraq	40,000
Isin, Iraq	40,000
Larsa, Iraq	40,000
Mari, Syria	30,000
Nippur, Iraq	30,000
Uruk, Iraq	30,000
Susa, Iran	25,000
Umma, Iraq	25,000
Ur, Iraq	20,000

Supplementary Table 3: Largest Cities in the Year 1000 BC

Largest Cities in the Year 1000 BC	Population
Thebes, Egypt	120,000
Babylon, Iraq	100,000
Memphis, Egypt	100,000
Loyang, China	50,000
Tanis, Egypt	35,000
Pyongyang, North Korea	25,000
Susa, Iran	25,000
Heliopolis, Egypt	20,000
Assur, Iraq	12,000
Nineveh, Iraq	10,000

Supplementary Table 4: Largest Cities in the Year AD 100

Largest Cities in the Year AD 100	Population
Rome, Italy	1,000,000
Alexandria, Egypt	500,000
Luoyang, China	420,000
Seleucia, Iraq	400,000
Antioch, Turkey	330,000
Carthage, Tunisia	250,000
Anuradha-Pura, Sri Lanka	200,000
Ephesus, Turkey	200,000
Capua, Italy	170,000
Taxilla, Pakistan	150,000

Supplementary Table 5: Largest Cities in the Year AD 1500

Largest Cities in the Year AD 1500	Population
Beijing, China	672,000
Vijayanagar, India	500,000
Cairo, Egypt	400,000
Hangzhou, China	250,000
Tabriz, Iran	250,000
Guar, India	200,000
Istanbul (Constantinople), Turkey	200,000
Paris, France	185,000
Guangzhou, China	150,000
Nanjing, China	147,000

Supplementary Table 6: Largest Cities in the Year AD 1800

Largest Cities in the Year AD 1800	Population
Beijing, China	1,100,000
London, England	861,000
Guangzhou, China	800,000
Tokyo (Edo), Japan	685,000
Istanbul (Constantinople),Turkey	570,000
Paris, France	547,000
Naples, Italy	430,000
Hangzhou, China	387,000
Osaka, Japan	383,000
Kyoto, Japan	377,000

Supplementary Table 7: Largest Cities in the Year AD 1975

Largest Cities in the Year AD 1975	Population
Tokyo (Edo), Japan	23,000,000
New York, USA	17,100,000
Osaka , Japan	15,500,000
Mexico City, Mexico	11,300,000
Moscow, Russia	10,700,000
London, England	10,500,000
Sao Paulo, Brazil	10,000,000
Paris, France	9,400,000
Los Angeles, USA	8,900,000
Buenos Aires, Argentina	8,400,000

Supplementary Table 8: Largest Cities in the Year AD 2000
The table below compares the largest cities in the year AD 2000 between the Modelski and *United Nations World Urbanization Prospects* (2014) datasets**.**

Largest Cities in the Year AD 2000	Population (Modelski)	Population (UN)
Montevideo, Uruguay	13,303,000	1,600,000
Seoul, South Korea	10,231,000	9,878,000
Sao Paolo, Brazil	10,009,000	17,014,000
Mumbai, India	9,926,000	16,367,000
Jakarta, Indonesia	9,373,000	8,390,000
Karachi, Pakistan	9,339,000	10,032,000
Moscow, Russia	8,297,000	10,005,000
Istanbul, Turkey	8,260,000	8,744,000
Mexico City, Mexico	8,235,000	18,457,000
Shanghai, China	8,214,000	13,959,000

Note: In **Table 8** above, Modelski's AD 2000 population estimates use UN *2000 Demographic Yearbook* data for "cities proper" and do not show urban agglomeration population data unless no other data were available. Please refer to Modelski's text for individual city exceptions. All Chinese cities show 1990 census results for Modelski's AD 2000 population estimates. The Population (UN) data uses *United Nations World Urbanization Prospects* (2014) for places with populations over 300,000 inhabitants.
